# Linkage study of 14 candidate genes and loci in four large Dutch families with vesico-ureteral reflux

**DOI:** 10.1007/s00467-007-0492-4

**Published:** 2007-08-01

**Authors:** Albertien M. van Eerde, Bobby P. C. Koeleman, Jiddeke M. van de Kamp, Tom P. V. M. de Jong, Cisca Wijmenga, Jacques C. Giltay

**Affiliations:** 1 Department of Medical Genetics KC.04.084.2, University Medical Centre Utrecht, P.O. Box 85090, 3508 AB Utrecht, The Netherlands; 2 Department of Pediatric Urology, University Medical Centre Utrecht, Utrecht, The Netherlands; 3 Department of Clinical Genetics, VU Medical Centre, Amsterdam, The Netherlands

**Keywords:** Vesico-ureteral reflux/genetics, Kidney diseases/genetics, Kidney diseases/pathology, Linkage (genetics)

## Abstract

Vesico-ureteral reflux (VUR) is a major contributing factor to end-stage renal disease in paediatric patients. Primary VUR is a familial disorder, but little is known about its genetic causes. To investigate the involvement of 12 functional candidate genes and two reported loci in VUR, we performed a linkage study in four large, Dutch, multi-generational families with multiple affected individuals. We were unable to detect linkage to any of the genes and loci and could exclude the *GDNF, RET, SLIT2, SPRY1, PAX2, AGTR2, UPK1A* and *UPK3A* genes and the 1p13 and 20p13 loci from linkage to VUR. Our results provide further evidence that there appears to be genetic heterogeneity in VUR.

**Electronic supplementary material:**

The online version of this article (doi:10.1007/s00467-007-0492-4) contains supplementary material, which is available to authorized users.

## Introduction

Vesico-ureteral reflux [VUR (MIM 193000)], the retrograde passage of urine from the bladder, is one of the most commonly detected congenital anomalies. With a prevalence of approximately 1% [1], VUR can be primary, due to an incompetent valve mechanism at the uretero-vesical junction, or secondary, due to a functional or anatomical urethral obstruction. VUR is often accompanied by non-neuropathic bladder/sphincter dysfunction (NNBSD). This complex is a major cause of urinary tract infections in children [2] and the sometimes resulting reflux nephropathy is the cause of approximately 7% of end-stage renal disease in paediatric patients in the Netherlands [3]. Severe primary VUR can concur with congenital renal insufficiency based on hypoplasia/dysplasia of one or both kidneys. Genetic factors play an important role in the aetiology of primary VUR, since siblings of affected children have a 32% risk of VUR [4], and since there is 80% concordance between monozygotic twins [5]. VUR may occur in isolation or as part of a syndrome, such as renal-coloboma syndrome. Apart from the recently published involvement of ROBO2 [6] little is known about the genetic causes of isolated primary VUR in humans. The aim of the present study was to confirm linkage to published candidate loci and genes. So far, only one genome-wide linkage study has been reported, which showed significant linkage to a 17 cM locus on chromosome 1p13 in five Caucasian families and suggestive linkage to chromosome 20p13 [7]. To date, these results have not been replicated [8]. Embryonal ectopic ureteral budding has been proposed to be a mechanism for the development of VUR [9, 10]. Defects of the *RET* and *GDNF* genes have been shown to cause ectopic ureteral budding [11, 12]. Hence, these and other genes involved in the *RET/GDNF* pathway are obvious functional candidate genes for VUR. Genes involved in syndromal VUR and genes derived from mouse models with urinary tract abnormalities (such as *AGTR2*) are also attractive functional candidate genes for VUR. The aim of this study was to assess the 1p13 and 20p13 loci and appropriate candidate genes (Table 1) for their role in the Dutch VUR population by performing linkage analysis in four large families.

**Table 1:** Genes tested in linkage study of four large multi-generational VUR families (*LOD* logarithm of the odds, *NPL* non-parametric linkage, *HLOD* heterogeneity LOD, *A* ureteral budding, *BRET/GDNF* pathway, *C* mouse and human phenotype, *D* linkage study, *E* in urothelial plaque with *UPK3A* (mouse model), *F* mouse model.)

Gene	Relevance	Chromosome	Location (cM)	Multi-point LOD score at the gene location	NPL	NPL *p*-value	Alpha^b^	HLOD^b^	Reference
*GDNF*	A/B	5	54	−2.03	0.70	0.22	0.10	0.01	[13]
*RET*	A/B	10	66	−2.55	−0.85	0.80	0.00	0.00	[13]
*SLIT2*	A/B	4	34	−2.15	0.35	0.32	0.15	0.80	[14]
*SPRY1*	A/B	4	126	−3.25	−0.98	0.86	0.00	0.00	[14]
*PAX2*	A/B	10	124	−3.43	−0.49	0.63	0.00	0.00	[13]
*AGTR2*	A/C	X	71	−3.81	−1.18	0.88	0.00	0.00	[15]
*HLADRB1*	D	6	46	−1.84	0.25	0.35	0.20	0.16	[16]
*UPK1A*	E	19	61	−2.90	−0.62	0.69	0.00	0.00	[17, 18]
*UPK1B*	E	3	138	0.15	1.24	0.12	0.65	0.43	[17, 18]
*UPK2*	E	11	115	−1.50	−0.22	0.52	0.00	0.00	[17, 18]
*UPK3A*	A/F	22	53	−3.40^a^	−1.20	0.80	0.00	0.00	[17, 18]
*UPK3B*	E	7	89	−1.08	0.59	0.25	0.00	0.00	[17, 18]

^a^Two-point analysis of marker D22S928; 0.5 cM away from *UPK3A*.

^b^Alpha: estimated proportion of families linked to result in corresponding heterogeneity LOD (HLOD). HLOD analyses were performed, but did not contribute and are not discussed.

## Methods

**Fig. 1:**
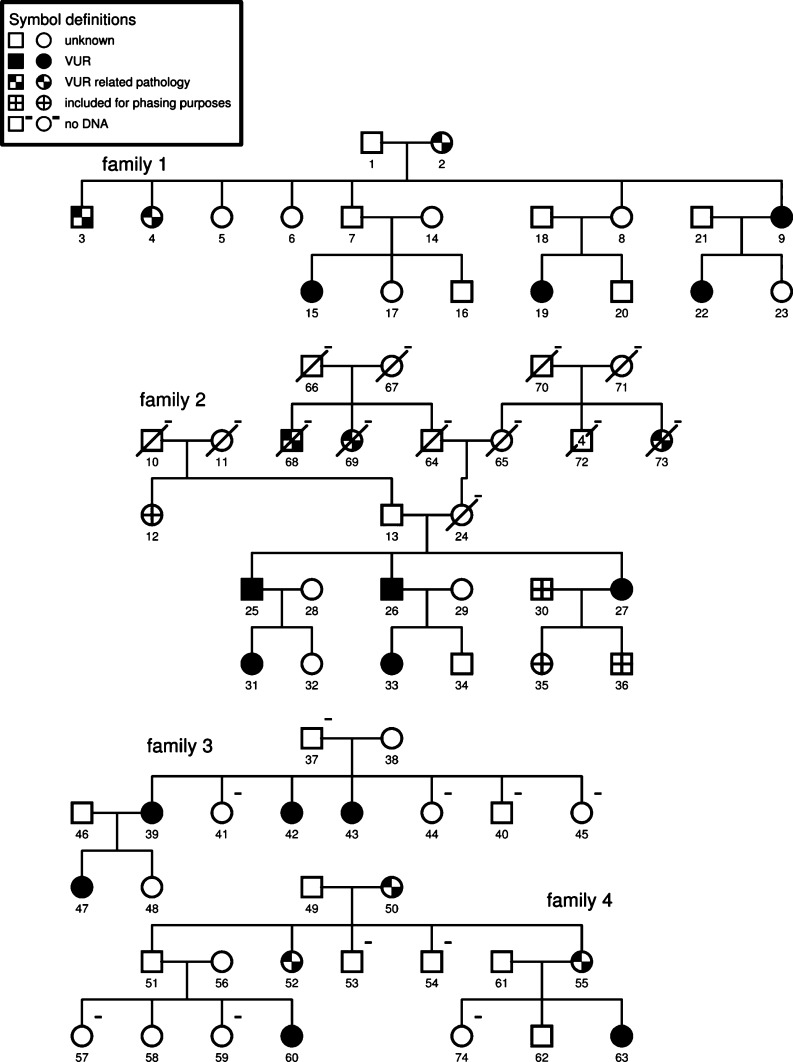
VUR family pedigrees (21 affected individuals in 51 samples). Family 1: *2* left kidney had to be removed at 5 years; *3, 4* end-stage renal disease (ESRD); *9* VUR and ESRD; *15* VUR and duplex collecting system; *19* VUR; *18* VUR and nephropathy. Family 2: *68, 69, 73* not included in analyses; *25, 26, 30* and *31* VUR; *33* VUR and dysfunctional voiding. Family 3: *39, 42, 43* VUR and dysfunctional voiding symptomatology; *47* VUR, dysfunctional voiding, meatal stenosis. Family 4: *50* and *52* recurrent urinary tract infections (UTIs) as a child, duplex collecting system; *55* UTIs and urinary tract operation; *49* VUR; *51* VUR and dysfunctional voiding symptomatology

DNA of four unrelated Dutch VUR families was collected (Fig. 1), which had been ascertained as part of a previous study [19]. Of a total of 51 samples there were 21 affected individuals. The families provided moderate power to detect linkage as calculated with SLINK (probability of obtaining LOD scores of at least 1.0, 2.0, or 3.0 was 74%, 49% and 18%, respectively). An affected phenotype for index patients was based on their having been treated for primary VUR, while for family members it was based on having a positive case history (of actual VUR, or multiple urinary tract infections with high fever as a child, or evidence of reflux nephropathy, such as requiring renal replacement therapy without obvious other causes) (see also Fig. 1). All other family members were classified as “unknown”, despite negative imaging results at a young age in some of them. Dutch paediatric urologists consider the use of voiding cysto-urethrography (VCUG) in asymptomatic children just for research purposes inappropriate. Therefore, we could not classify family members as “not affected”.

Some of the candidate genes play roles in congenital anomalies of the kidney and urinary tract (CAKUT) phenotypes (such as VUR, duplex collecting system and renal hypoplasia in mice) [9, 15]. Therefore, families in which one or more patients had these kinds of phenotypes (and VUR) were not excluded. All participants gave their informed consent, and the Medical Ethics Committee of the University Medical Centre Utrecht approved the study.

For the 1p13 locus, we started out with the markers flanking the reported 1p13 linkage peak (D1S1653 and GATA176C01) [7]. Both markers now have different map locations if one is reviewing the most recent updates of the Ensembl (v38) and Marshfield databases. In fact, the telomeric marker GATA176C01 (D2S2972) even maps to a different chromosome (2q11). The centromeric marker D1S1653, which in our query result has roughly the same genetic position (164.09-166 cM) as previously published [7, 8], localizes on chromosome 1q23. Therefore, we tested both the entire 1p13–1q23 and 2q11 loci for linkage to VUR. A total of 11 short tandem repeat polymorphism (STRP) markers for 1p13–1q23 and seven STRP markers for 2q11 (with an average intermarker distance of 5 cM) were chosen to saturate the regions spanning 55.3 Mb on chromosome 1 and 46.3 Mb on chromosome 2. For 20p13, five STRP markers were selected, spanning 12.0 Mb (Supplementary Table 1 Online).

For the candidate genes, we aimed to cover the specific location with an average intermarker distance of 2 cM (Supplementary Table 1 Online).

Markers were genotyped as described elsewhere [20] in the 51 family members, together with three Centre d’Etude Polymorphism Humaine (CEPH) reference samples and three negative controls. The polymerase chain reactions (PCRs) were carried out on a GeneAmp PCR system 9700 machine (Applied Biosystems). The PCR products were separated on an ABI 3730 DNA sequencer (Applied Biosystems). The output was analysed with Genemapper 3.5 software (Applied Biosystems). Two investigators checked all the genotypes, and we verified the identity of the markers by comparing genotypes of the CEPH reference samples with the CEPH genotype database. A Mendelian inheritance check was performed with PedCheck 1.1 software [21], and samples with Mendelian errors were excluded from the linkage analysis.

Multi-point (both parametric and non-parametric) analyses were performed for all markers with GENEHUNTER (version 2.1_r2 beta), or GENEHUNTER PLUS (for X-linked dominant calculations in *AGTR2*) [22]. We assumed an autosomal dominant model with reduced penetrance (0.8) for the parametric analyses, similar to the parameters previously described [7, 23]. This mode of inheritance agreed most with our pedigrees (Fig. 1). The phenocopy rate was equal to the population frequency of VUR (0.01). Disease allele frequency was assumed to be 0.01. Regions with a parametric LOD score ≤ −2 were defined as exclusion regions [24]. All significance levels applied in this study were based on previously proposed thresholds [24, 25].

## Results

Twelve functional candidate genes were screened for linkage to VUR. The multi-point LOD score obtained for each of the 12 genes (at the genetic location of the gene) is shown in Table 1, together with the non-parametric linkage (NPL) score and corresponding *P* value. Multi-point LOD scores with NPL score and corresponding *P* value for all markers are shown in Supplementary Table 1 Online. Eight of the functional candidate genes (*GDNF, RET, SLIT2, SPRY1, PAX2, AGTR2, UPK1A* and *UPK3A*) were completely excluded. For the other four, the results were inconclusive, although linkage is highly unlikely.

**Fig. 2:**
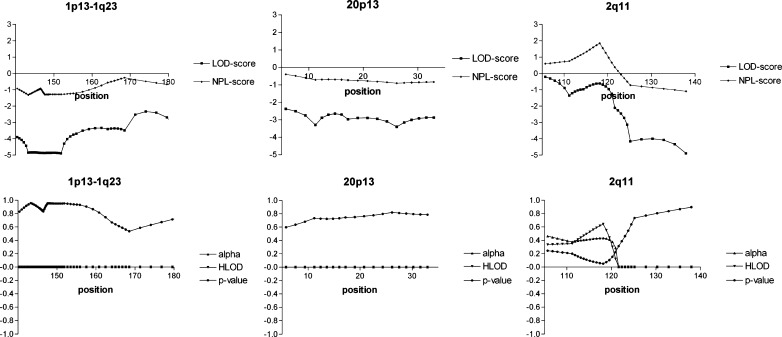
Multi-point LOD plots for the reported loci tested in the linkage study of four large multi-generational VUR families. Because one of the flanking markers of the 1p13 locus proved to actually map to chromosome 2, we also tested the chromosome 2q11 region. HLOD (heterogeneity LOD) analyses did not contribute. Positions in cM

For the reported linkage regions, no significant linkage was detected either. One of the markers reported to be on chromosome 1 [7] appeared to reside on chromosome 2 (see Methods section). Therefore, both the original locus on chromosome 1p13 and the “new” locus on chromosome 2q11 were tested. The chromosome 1 locus was completely excluded, as the multi-point LOD score was below −2 for the entire region. Sixty-one percent of the locus on chromosome 2 could be totally excluded. The locus on chromosome 20p13 was completely excluded (Fig. 2).

## Discussion

We performed a comprehensive screen of 12 functional candidate genes and two reported loci (which later proved to be three separate regions). All the genes, except *HLADRB1*, had, in some way, been proven to play a role (indirectly) in ectopic ureteral budding and were thought likely to play a role in human primary VUR. However, we did not detect linkage to any of them. We were able to exclude eight genes (*GDNF, RET, SLIT2, SPRY1, PAX2, AGTR2, UPK1A* and *UPK3A*) as major players in these Dutch VUR families. *ROBO2*, the receptor of *SLIT2* [26], had already been ruled out, since it had been sequenced in the four probands in a parallel study; no mutations were detected [6], therefore it was not included in the present study. Nevertheless, these genes may still be involved in the mechanism causing VUR. The moderate power these pedigrees provided to reveal linkage means that it is possible that the genes that showed inconclusive results might have yielded positive results in a more highly powered study. Since the *RET/GDNF* pathway plays such a central role in ureteral budding, it is plausible that these genes are, indeed, causative factors for VUR but that the pathogenetic variants lie in upstream or downstream regulatory elements. Other genes that are more or less directly involved in the *RET/GDNF* pathway or in ureteral budding in general, such as *EYA1, GATA3, WT1* or *BMP4* [27], may also contribute to VUR.

We could not confirm linkage for any of the reported loci, and we were even able to exclude completely the 1p13 and 20p13 loci. This is the second non-replication of the 1p13 region [8]. Our data show that the 1p13 locus resides either on chromosome 1p13 to 1q23 or on 2q11. We also excluded linkage to most of this 2q11 locus.

We realize that these families show some intra-familial and inter-familial heterogeneity. This heterogeneity, however, is similar to that described in VUR families by others [7]. In older generations (Fig. 1) it is impossible to have more data than self-reported history, but the described phenotypes are very likely to be caused by VUR. Therefore, we did assign the affected status to these patients, but we are aware of the fact that this is one of the limitations of the study.

One of the major obstacles for linkage studies in VUR is the relative rarity of large pedigrees, which is due to many children growing out of the disorder, the reduced penetrance of the trait, and the locus heterogeneity [8]. Furthermore, when doing linkage studies in relatively few families, one assumes a large effect of one or few genes. Maybe the genes of interest do play a role, but their effect is too small to be picked up. Therefore, studies like ours and those published [7, 8] are useful to search for one or more major genes. For this reason, association studies with large sample sizes may offer a better approach for unravelling the genetics of VUR. Both a hypothesis-free (genome-wide) approach and a more elaborate candidate gene study would be interesting follow-up studies. Alternatively, it might be interesting to study the role of copy number variants (CNVs) in VUR, since, recently, such CNVs were proposed to be involved in the mechanism underlying a number of complex disorders [28, 29]. Apart from those in a recent study by Lu et al. [6], no genes have been published that appear to be directly involved in primary VUR in humans, and no replication of the linkage peak on 1p13 [7] has been reported. Our results provide further evidence for genetic heterogeneity in VUR. We hypothesize that several genes, which still have to be identified but which are likely to affect ureteral budding, will each play a role in the pathogenesis of VUR.

## Electronic supplementary material

Below is the link to the electronic supplementary material.

Supplementary Table 1 Online Markers used in the linkage study of four large, Dutch, multi-generational VUR families. *MPL* multi-point LOD score (DOC 148 kb)

## References

[1] Grand Round (1996). Vesicoureteric reflux: all in the genes? Report of a meeting of physicians at the Hospital for sick children, Great Ormond Street, London. Lancet.

[2] van Gool JD, Hjalmas K, Tamminen-Mobius T, Olbing H (1992). Historical clues to the complex of dysfunctional voiding, urinary tract infection and vesicoureteral reflux. The international reflux study in children. J Urol.

[3] Miklovicova D, Cornelissen M, Cransberg K, Groothoff JW, Dedik L, Schroder CH (2005). Etiology and epidemiology of end-stage renal disease in Dutch children 1987–2001. Pediatr Nephrol.

[4] Hollowell JG, Greenfield SP (2002). Screening siblings for vesicoureteral reflux. J Urol.

[5] Kaefer M, Curran M, Treves ST, Bauer S, Hendren WH, Peters CA, Atala A, Diamond D, Retik A (2000). Sibling vesicoureteral reflux in multiple gestation births. Pediatrics.

[6] Lu W, van Eerde AM, Fan X, Quintero-Rivera F, Kulkarni S, Ferguson H, Kim HG, Fan Y, Xi Q, Li QG, Sanlaville D, Andrews W, Sundaresan V, Bi W, Yan J, Giltay JC, Wijmenga C, de Jong TP, Feather SA, Woolf AS, Rao Y, Lupski JR, Eccles MR, Quade BJ, Gusella JF, Morton CC, Maas RL (2007). Disruption of ROBO2 is associated with urinary tract anomalies and confers risk of vesicoureteral reflux. Am J Hum Genet.

[7] Feather SA, Malcolm S, Woolf AS, Wright V, Blaydon D, Reid CJ, Flinter FA, Proesmans W, Devriendt K, Carter J, Warwicker P, Goodship TH, Goodship JA (2000). Primary, nonsyndromic vesicoureteric reflux and its nephropathy is genetically heterogeneous, with a locus on chromosome 1. Am J Hum Genet.

[8] Sanna-Cherchi S, Reese A, Hensle T, Caridi G, Izzi C, Kim YY, Konka A, Murer L, Scolari F, Ravazzolo R, Ghiggeri GM, Gharavi AG (2005). Familial vesicoureteral reflux: testing replication of linkage in seven new multigenerational kindreds. J Am Soc Nephrol.

[9] Ichikawa I, Kuwayama F, Pope JCt, Stephens FD, Miyazaki Y (2002). Paradigm shift from classic anatomic theories to contemporary cell biological views of CAKUT. Kidney Int.

[10] Mackie GG, Stephens FD (1975). Duplex kidneys: a correlation of renal dysplasia with position of the ureteral orifice. J Urol.

[11] Moore MW, Klein RD, Farinas I, Sauer H, Armanini M, Phillips H, Reichardt LF, Ryan AM, Carver-Moore K, Rosenthal A (1996). Renal and neuronal abnormalities in mice lacking GDNF. Nature.

[12] Schuchardt A, D’Agati V, Pachnis V, Costantini F (1996). Renal agenesis and hypodysplasia in ret-k- mutant mice result from defects in ureteric bud development. Development.

[13] Woolf AS, Winyard PJ (2002). Molecular mechanisms of human embryogenesis: developmental pathogenesis of renal tract malformations. Pediatr Dev Pathol.

[14] Murawski IJ, Gupta IR (2006). Vesicoureteric reflux and renal malformations: a developmental problem. Clin Genet.

[15] Nishimura H, Yerkes E, Hohenfellner K, Miyazaki Y, Ma J, Hunley TE, Yoshida H, Ichiki T, Threadgill D, Phillips JA, Hogan BM, Fogo A, Brock JW, Inagami T, Ichikawa I (1999). Role of the angiotensin type 2 receptor gene in congenital anomalies of the kidney and urinary tract, CAKUT, of mice and men. Mol Cell.

[16] Kawauchi A, Takahara S, Sada M, Goto R, Nakatani T, Miki T (2001). Susceptibility to vesicoureteral reflux in Japanese is linked to HLA-DR antigen. Urology.

[17] Deng FM, Liang FX, Tu L, Resing KA, Hu P, Supino M, Hu CC, Zhou G, Ding M, Kreibich G, Sun TT (2002). Uroplakin IIIb, a urothelial differentiation marker, dimerizes with uroplakin Ib as an early step of urothelial plaque assembly. J Cell Biol.

[18] Hu P, Deng FM, Liang FX, Hu CM, Auerbach AB, Shapiro E, Wu XR, Kachar B, Sun TT (2000). Ablation of uroplakin III gene results in small urothelial plaques, urothelial leakage, and vesicoureteral reflux. J Cell Biol.

[19] Giltay JC, van de Meerakker J, van Amstel HK, de Jong TP (2004). No pathogenic mutations in the uroplakin III gene of 25 patients with primary vesicoureteral reflux. J Urol.

[20] Verbeek DS, Schelhaas JH, Ippel EF, Beemer FA, Pearson PL, Sinke RJ (2002). Identification of a novel SCA locus (SCA19) in a Dutch autosomal dominant cerebellar ataxia family on chromosome region 1p21-q21. Hum Genet.

[21] O’Connell JR, Weeks DE (1998). PedCheck: a program for identification of genotype incompatibilities in linkage analysis. Am J Hum Genet.

[22] Kruglyak L, Daly MJ, Reeve-Daly MP, Lander ES (1996). Parametric and nonparametric linkage analysis: a unified multipoint approach. Am J Hum Genet.

[23] Chapman CJ, Bailey RR, Janus ED, Abbott GD, Lynn KL (1985). Vesicoureteric reflux: segregation analysis. Am J Med Genet.

[24] Edwards JH (1987). Exclusion mapping. J Med Genet.

[25] Lander E, Kruglyak L (1995). Genetic dissection of complex traits: guidelines for interpreting and reporting linkage results. Nat Genet.

[26] Grieshammer U, Le M, Plump AS, Wang F, Tessier-Lavigne M, Martin GR (2004). SLIT2-mediated ROBO2 signaling restricts kidney induction to a single site. Dev Cell.

[27] Bouchard M (2004). Transcriptional control of kidney development. Differentiation.

[28] Redon R, Ishikawa S, Fitch KR, Feuk L, Perry GH, Andrews TD, Fiegler H, Shapero MH, Carson AR, Chen W, Cho EK, Dallaire S, Freeman JL, Gonzalez JR, Gratacos M, Huang J, Kalaitzopoulos D, Komura D, MacDonald JR, Marshall CR, Mei R, Montgomery L, Nishimura K, Okamura K, Shen F, Somerville MJ, Tchinda J, Valsesia A, Woodwark C, Yang F, Zhang J, Zerjal T, Zhang J, Armengol L, Conrad DF, Estivill X, Tyler-Smith C, Carter NP, Aburatani H, Lee C, Jones KW, Scherer SW, Hurles ME (2006). Global variation in copy number in the human genome. Nature.

[29] Wilson GM, Flibotte S, Chopra V, Melnyk BL, Honer WG, Holt RA (2006). DNA copy-number analysis in bipolar disorder and schizophrenia reveals aberrations in genes involved in glutamate signaling. Hum Mol Genet.

[30] Broman KW, Murray JC, Sheffield VC, White RL, Weber JL (1998). Comprehensive human genetic maps: individual and sex-specific variation in recombination. Am J Hum Genet.

