# Supplementary material for: Linkage study of 14 candidate genes and loci in four large Dutch families with vesico-ureteral reflux
Source: Pediatr Nephrol. 2007 Aug 1;22(8):1129–33. doi: 10.1007/s00467-007-0492-4 (PMC1915619; doi:10.1007/s00467-007-0492-4)
Supplement: Supplementary file 1 — Markers used in the linkage study of four large, Dutch, multi-generational VUR families. MPL multi-point LOD score (DOC 148 kb) [file 467_2007_492_MOESM1_ESM.doc]

Supplementary table 1 online.
Markers used in linkage study of four large, Dutch, multi-generational VUR families. MPL: Multi-point LOD score.

| Locus/gene | Chromosome | Marker | Position (cM) | Position (bp) | Forward primer sequence | Reverse primer sequence | MPL | MPL fam 1 | MPL fam 2 | MPL fam 3 | MPL fam 4 | NPL | NPL *P*-value |
| --- | --- | --- | --- | --- | --- | --- | --- | --- | --- | --- | --- | --- | --- |
| 1p13-1q23 | 1 | D1S3723 | 140.39 | b | ATCTCATCAAGGTCAAATCCC | TTAGCATCCAATCAGAGAAGC | -3.89 | -0.15 | -1.12 | -0.92 | -1.71 | -0.92 | 0.829 |
|  | 1 | D1S2695 | 143.31 | 110497630 | GGAGGGCACTGGCTACA | TGCTGGCTCAGGGGAC | -4.85 | -0.14 | -2.04 | -0.88 | -1.79 | -1.31 | 0.957 |
|  | 1 | D1S187 | 145.45 | 112162152 | AGGTGTGAGCTGTTCTCATA | GCAAGACAGCTGCCTCATA | -4.84 | -0.12 | -2.01 | -0.92 | -1.79 | -1.06 | 0.883 |
|  | 1 | D1S502 | 146.53 | b | GGGTCACCTCTGAGGA | CTGGGCAAAAAGTGGA | -4.87 | -0.12 | -2.04 | -0.92 | -1.79 | -0.93 | 0.831 |
|  | 1 | D1S2746 | 147.60 | 112827308 | TAGCCTGGGCAACATAGATA | CTGGGAGCTCTATTCTGTCC | -4.88 | -0.14 | -2.02 | -0.92 | -1.79 | -1.30 | 0.954 |
|  | 1 | D1S1675 | 149.20 | 114541676 | CTAGCCAAGGCAGGTCTGTA | GCCTAGACAATGGGAGAGGT | -4.86 | -0.15 | -2.03 | -0.90 | -1.78 | -1.28 | 0.950 |
|  | 1 | D1S534 | 151.88 | 119479787 | AGCACATAGCAGGCACTAGC | CGATTGTGCCACTACACAGT | -4.89 | -0.16 | -2.02 | -0.93 | -1.78 | -1.28 | 0.951 |
|  | 1 | D1S2612 | 155.89 | 146252391 | GCTGTTCTTAGGGCTTTTCC | AACTTGGGCTTCTCTGCTTC | -3.69 | -0.4 | -1.13 | -0.92 | -1.24 | -1.20 | 0.931 |
|  | 1 | D1S1653 | 164.09 | 156199398 | GGAAAGCCTGTAGGAAGAGG | CCTGGATGACAGAGTGCTCT | -3.41 | -0.35 | -1.61 | -0.92 | -0.53 | -0.57 | 0.666 |
|  | 1 | D1S1167 | 168.52 | 158275688 | TCTGGGGGCTTAGAATAGAC | TCAGTGTGTAGGGACAAGATG | -3.49 | -0.11 | -1.99 | -0.93 | -0.46 | -0.26 | 0.534 |
|  | 1 | D1SDVCAG1^a^ | 182.4 | 165776203 | GTAGAAGCACATGGTGTGG | CTGGAGTAAATGCAGCTCAG | -3.48 | -0.68 | -1.12 | -0.83 | -0.86 | -0.76 | 0.756 |
| 20p13 | 20 | D20S103 | 2.13 | 507263 | GTTCATAGAGGGACAAGACACAGT | CCATGATGTTTGGTTAATCACA | -2.37 | -0.19 | -0.65 | -0.78 | -0.75 | -0.26 | 0.537 |
|  | 20 | D20S116 | 11.20 | 4001387 | TGACCACAGGGGTTAATG | CAGGACTCAGTGCACCAG | -3.30 | -0.42 | -1.10 | -0.93 | -0.85 | -0.72 | 0.733 |
|  | 20 | D20S905 | 17.19 | 5811629 | AGCTTGAGGAGCAGTGTCTT | TCAGCAGATCCCACCA | -2.97 | -0.38 | -1.10 | -0.64 | -0.84 | -0.74 | 0.744 |
|  | 20 | D20S901 | 26.13 | 9989532 | CTACTGCGCTCCATGAGA | CTGGTTGGTGGTCAACATA | -3.41 | -0.38 | -1.11 | -1.12 | -0.80 | -0.91 | 0.819 |
|  | 20 | D20S604 | 32.94 | 12532311 | TGAGTGAATTTTTCTAATAAATCCC | AAGCAATCTCATTTATTTTTACACA | -2.88 | -0.36 | -1.15 | -0.51 | -0.85 | -0.83 | 0.785 |
| 2q11 | 2 | D2S1790 | 105.7 | 84928800 | ACATGTCGATCTCAGCGTTC | GAGTTTTATTGGCCAAAGCA | -0.21 | -0.92 | -0.42 | 0.41 | 0.72 | 0.59 | 0.245 |
|  | 2 | D2S113 | 111.21 | 96641286 | GCTTGTTTCATCTCACCCTG | CTGTTGTTTTTTAGGTGGGAG | -1.36 | -1.53 | -1.13 | 0.49 | 0.80 | 0.76 | 0.198 |
|  | 2 | D2S2972 | 114.42 | 101938508 | TCTCACTACACCACCTGGGT | CTCTATCTCTATCTCTGTCTCTGCC | -0.97 | -1.31 | -1.13 | 0.51 | 0.96 | 1.21 | 0.109 |
|  | 2 | D2S436 | 118.16 | b | GATATGGGAGCAACATGAGC | GGAATCAACTTTCAGTATAAACCC | -0.62 | -1.12 | -1.13 | 0.52 | 1.11 | 1.85 | 0.049 |
|  | 2 | D2S1888 | 121.63 | 111111607 | TTTGAAGTTTGGTGTCTGTG | TGAAGTCCCTTGGAAATGTT | -2.11 | -1.09 | -1.12 | 0.48 | -0.39 | 0.37 | 0.312 |
|  | 2 | D2S410 | 125.18 | 115957399 | ATGTAGAAGAGTCAGAATTTCAACC | CAGACACAAATGCACACACA | -4.16 | -1.77 | -1.09 | -0.91 | -0.39 | -0.72 | 0.733 |
|  | 2 | D2S1260 | 137.93 | 131307274 | AGGTGAGATGGTGCCGCTG | CAGGAGGAGGCAATGCCCA | -4.89 | -1.99 | -1.12 | -0.93 | -0.85 | -1.09 | 0.896 |
| GDNF | 5 | D5S2023 | 53.67 | 37349306 | GAGCACTTGAGCCTGG | CATAAAATGAAGTAGGAGTGGTGTA | -2.15 | -1.98 | -0.69 | 0.53 | 0.00 | 0.69 | 0.215 |
|  | 5 | D5S1964 | 54.79 | 37895179 | TGTGACACAAAAGCACACAG | TTATCACCAAATAGGCAGGA | -1.80 | -1.75 | -0.58 | 0.53 | 0.00 | 0.70 | 0.214 |
| *RET* | 10 | D10S1793 | 68.63 | 49806348 | AGGGGTCACGGTCCAT | CTGCTTCCCAAGGTCAGG | -3.09 | -0.37 | -0.92 | -0.93 | -0.87 | -0.94 | 0.830 |
|  | 10 | D10S1220 | 70.23 | 52348570 | GATTGCTTTAGAAGTGGGCA | TGTAGGTTTTCACATGTTCAGC | -3.12 | -0.37 | -1.12 | -0.75 | -0.87 | -1.02 | 0.864 |
| *SLIT2* | 4 | D4S2946 | 33.42 | 17362511 | GTCAAGAGGGCTGATTCTG | ACCTGTCTGAACTTGCGTG | -2.34 | -1.39 | 0.82 | -0.93 | -0.84 | 0.33 | 0.326 |
|  | 4 | D4S2397 | 42.74 | 26866965 | CATGCACACCAAAACAAGAA | GCAACAAACCTGCACATTCT | -0.57 | -0.84 | 0.76 | 0.11 | -0.60 | 0.66 | 0.227 |
| *SPRY1* | 4 | D4S1612 | 124.45 | 122544018 | AAGGCTTTATTCNCTTATTGTT | GGTCCAAAGACAGGTCAAA | -3.21 | -1.39 | -1.32 | 0.00 | -0.50 | -0.95 | 0.841 |
|  | 4 | D4S430 | 126.15 | 123827098 | CTGTATATGTTAATGTGC | GGACCCAGTCTTGCTATG | -3.25 | -1.28 | -1.34 | 0.00 | -0.64 | -0.98 | 0.863 |
|  | 4 | D4S1615 | 128.31 | 128429186 | CCTTGGGTCAGCCACATATC | CACTCAGAACAGAAACTTGGGT | -3.08 | -1.27 | -0.85 | 0.00 | -0.95 | -1.05 | 0.887 |
| *PAX2* | 10 | D10S1709 | 119.70 | 99475362 | GTGAGTCCAGAATCACCCC | CAGTGGAATGGCTCATTTG | -3.20 | -0.21 | -2.05 | -0.93 | -0.02 | -0.45 | 0.614 |
|  | 10 | D10S184 | 121.98 | 100121771 | AAAATGAGGGAAGTTGGGA | TCTTTTCTTGCCTCTTCCAT | -3.32 | -0.32 | -1.97 | -0.93 | -0.10 | -0.45 | 0.615 |
|  | 10 | D10S1239 | 125.41 | 103186343 | CCCTAGCTAATGTTAAAATATCACG | CAGAGTGAGACCCTGTTTCA | -3.59 | -0.39 | -1.90 | -0.91 | -0.39 | -0.52 | 0.645 |
| *AGTR2* | X | DXS6797 | 67.12 | 107367721 | TTCCCTCTCTCCCTCTGTCT | ACACACACCCAAAACCAGAT | -4.20 | -0.62 | -1.41 | -1.08 | -1.09 | -1.34 | 0.929 |
|  | X | DXS6804 | 68.74 | 111999363 | CCCAGATATTTTGACCACCA | GGCATGTGGTTGCTATAACC | -4.04 | -0.62 | -1.24 | -1.08 | -1.10 | -1.34 | 0.929 |
|  | X | DXS1001 | 75.79 | 119720696 | TACAAGTAACCCTCGTGACA | GTTATGGAATCAATCCAAGTG | -3.72 | -0.13 | -1.41 | -1.08 | -1.10 | -0.86 | 0.804 |
| *HLADRB1* | 6 | D6S1281 | 44.41 | 25405006 | GATGCCACGTTTTAAAATGC | AGAAGCAGCTGTGCTTTGTT | -1.79 | -0.4 | -1.05 | -1.30 | 0.96 | 0.35 | 0.321 |
|  | 6 | D6S291 | 49.50 | 36373494 | CTCAGAGGATGCCATGTCTAAAATA | GGGGATGACGAATTATTCACTAACT | -2.64 | -1.12 | -1.12 | -1.31 | 0.90 | -0.05 | 0.455 |
| *UPK1A* | 19 | D19S220 | 62.03 | 43123394 | GTGTCTTATGTTCAGAAAGGCCATGTCATTTG | TCCCTAACGGATACACAGCAACAC | -3.30 | -0.37 | -2.25 | -0.11 | -0.56 | -0.65 | 0.703 |
|  | 19 | D19S400 | 64.70 | 46219373 | CGGTATGTCTTTATCAGCAG | ATGACAGCTCTAGGAAGGC | -4.30 | -0.38 | -1.88 | -0.93 | -1.11 | -0.93 | 0.825 |
| *UPK1B* | 3 | D3S3665 | 129.73 | 115686173 | GGGCCTCAAAGCACTTC | CCATCTTCAGGCAGTAAAGC | 0.65 | -0.82 | 0.66 | 0.53 | 0.28 | 1.80 | 0.056 |
|  | 3 | D3S2460 | 134.64 | 118885068 | ATTGCTTCCCCTTTACCTGA | GACAGGAGACAGAATGTTATAAGTT | 0.33 | -1.08 | 0.62 | 0.54 | 0.26 | 1.65 | 0.067 |
|  | 3 | D3S3606 | 143.94 | 128682900 | AAAATTCCCTGCAGTGGGA | GGGGCTCGAAAGACAGTAAA | -2.14 | -1.23 | -1.01 | 0.48 | -0.39 | 0.54 | 0.257 |
| *UPK2* | 11 | D11S4104 | 113.93 | 118140629 | GGAGAATGGCCTGAACCTG | ATCTCTATCATGGGCAATTTGG | -1.63 | -0.24 | -0.56 | 0.02 | -0.85 | -0.11 | 0.477 |
|  | 11 | D11S924 | 115.53 | 118943143 | ATTGAACTCCAGCCCG | CCTCATTGGGCCACTC | -1.46 | -0.23 | -0.50 | -0.03 | -0.70 | -0.28 | 0.539 |
| *UPK3A* | 22 | D22S928 | 52.08 | 43854113 | TGCAAAGTGCTGGAGG | TGAAGATGGCTAGTACGGG | -3.66 | -0.49 | -0.93 | -1.12 | -1.12 | -1.23 | 0.923 |
| *UPK3B* | 7 | D7S1870 | 86.12 | 73764649 | TTCACTCAGGAAGTGGC | TGGTGATGTGCTTTACTACG | -1.25 | -0.91 | 0.00 | 0.48 | -0.82 | 0.38 | 0.309 |
|  | 7 | D7S2204 | 90.95 | 77964859 | TCATGACAAAACAGAAATTAAGTG | AGTAAATGGAATTGCTTGTTACC | -1.03 | -1.02 | 0.00 | 0.37 | -0.38 | 0.75 | 0.199 |

^a^ self-designed marker,

^b^ is not mapped to the assembly in Ensembl v38.
